# Effectiveness of antiseptics in the bacterial load reduction after septic wound dressing at Bugando Medical Centre, Mwanza, Tanzania

**DOI:** 10.11604/pamj.2025.52.108.41462

**Published:** 2025-11-13

**Authors:** Emmanuel Jagadi, Doris George Makweta, Magwa Jisusi Kiyumbi, Helmut Nyawale, Zengo Kashinje, Vitus Silago, Francis Tegete, Inyasi Lawrence Akaro, Jeremiah Seni

**Affiliations:** 1 Archbishop Anthony Mayalla School of Nursing, Catholic University of Health and Allied Sciences, Mwanza, Tanzania,; 2 School of Pharmacy, Catholic University of Health and Allied Sciences, Mwanza, Tanzania,; 3 Department of Microbiology and Immunology, Weill-Bugando School of Medicine, Catholic University of Health and Allied Sciences, Mwanza, Tanzania,; 4 Department of Surgery, Weill Bugando School of Medicine, Catholic University of Health and Allied Sciences, and Bugando Medical Centre, Mwanza, Tanzania,; 5 Department of Orthopaedic Surgery, Weill Bugando School of Medicine, Catholic University of Health and Allied Sciences, and Bugando Medical Centre, Mwanza, Tanzania

**Keywords:** Septic wounds, antiseptics effectiveness, Bugando, Tanzania

## Abstract

**Introduction:**

septic wounds due to multidrug-resistant bacteria (MDR) are costly and result in adverse patient outcomes. Despite the fact that various antiseptics are routinely used for wound dressing, their effectiveness on bacterial load reduction remains to be evaluated to ascertain the usefulness of this step prior to antimicrobial therapies.

**Methods:**

a cross-sectional analytical study was conducted among 203 patients with septic wounds at Bugando Medical Centre (BMC). Wound swab samples before and after dressing were collected, and cultured to quantify the total bacteria, extended-spectrum beta-lactamase-producing (ESBL) Gram-negative bacteria, and Methicillin-resistant Staphylococcus aureus (MRSA) load reduction.

**Results:**

half of the wounds were due to road traffic accidents. A total of 146 (71.9%) patients had positive aerobic culture before wound dressing, which decreased to 39.4% (80/203) after dressing (p-value < 0.001). The median (IQR) of total bacterial load before and after dressing was 205.5 (112-330) and 128 (34.5-235) CFU/mL, respectively (p-value<0.001). The proportions of ESBL-producing Gram-negative bacteria and MRSA were 34.0% and 39.9%, respectively, before dressing (and significantly reduced to 20.2% and 11.3%, respectively, after dressing). Povidone-iodine was independently associated with decreased odds of culture positivity after wound dressing [OR: 95% CI = 0.09 (0.01-0.63), p-value =0.016].

**Conclusion:**

bacterial culture positivity was halved after septic wound dressing. Povidone-iodine significantly reduced both the total bacteria and MDR bacterial load in these wounds. Routine monitoring of antiseptics´ effectiveness is reiterated as a pivotal pre-requisite step prior to antibiotic therapies in septic wound management.

## Introduction

Septic wounds are characterized by pus, excavation of the skin or underlying soft tissue, and are most often caused by bacteria or fungal pathogens [1,2]. Infected wounds are associated with profound discomfort, prolonged hospital stay due to delayed recovery, and enormous health care expenditures when implicated pathogens are multi-drug resistant bacteria (MDR) [3-5]. Globally, surgical site infections (SSIs) and non-healing or chronic wounds substantially contribute to healthcare-associated infections (HAI) and community-associated infections (CAI), respectively [6,7]. Although SSIs are low in developed countries compared to developing countries [6], the overall prevalence of SSIs post-caesarian sections and among general surgical patients has remained relatively higher (10.0% and 26%, respectively) at Bugando Medical Center (BMC) tertiary hospital in Tanzania [8,9]. In this hospital, bacteria colonizing chronic lower limb ulcers are commonly MDR, with predominance of two phenotypes, namely Methicillin-resistant *Staphylococcus aureus* (MRSA) in approximately 44.4% and Extended-spectrum beta-lactamases (ESBL) producing Gram-negative bacteria in approximately 35.0% [10]. Therefore, searching for rational antimicrobial therapies and other management modalities for septic wounds remains an ongoing challenge among health care professionals. Antiseptics are chemical agents that kill or inhibit the multiplication of microorganisms, and are essential first-line chemical agents for wound management [11,12].

Antiseptics are prerequisites for the reduction of bacterial load in septic wounds prior to antibiotic administration. The most commonly used antiseptics in clinical practice include povidone-iodine, polyhexamethylene biguanide, chlorhexidine, alcohol, hydrogen peroxide, boric acid, silver nitrate, silver sulfadiazine, and sodium hypochlorite [11,12]. Their spectrum of activities varies depending on their chemical constituents, concentration of the active ingredients, inanimate or animate surfaces targeted, and other environmental conditions [12]. Despite extensive research on the causes of wound infections, the use of standardized surgical protocols/procedures and antibiotic therapeutic options for septic wound management, the role of antiseptics in the total bacterial load reduction has been sparsely studied [1,13]. This study had three objectives. Firstly, to evaluate the effectiveness of antiseptics in the total bacterial load reduction and MDR bacterial load reduction (i.e. ESBL producing Gram- negative bacteria and MRSA) after septic wounds to guide evidence-based infection prevention and control measures (IPC). Secondly, to determine bacterial species implicated in causing wound infections, and thirdly, to determine factors associated with bacterial load reduction after septic wound dressing at BMC Mwanza - Tanzania.

## Methods

**Study design:** this was a cross-sectional analytical study.

**Settings:** this study was conducted from May to August 2021. The BMC is a tertiary hospital in the northwestern part of Tanzania with approximately 1000 beds capacity. It attends around 300,000 patients each year and serves as a referral hospital for eight regions, which altogether constitute a catchment population of approximately 20 million people. Bugando Medical Centre is also a teaching hospital for the Catholic University of Health and Allied Sciences (CUHAS).

**Participants:** this study included patients (outpatients and inpatients) with septic wounds of all age groups attending surgical departments at BMC. Patients with septic wounds who were already enrolled in the study but were attending for subsequent hospital visits, and those who did not consent to the study, were excluded.

**Variables:** outcome variables in this study included culture positivity after wound dressing, bacterial load reduction after wound dressing, and AMR bacterial phenotypes (MRSA and ESBL-producing Gram-negative bacteria) after wound dressing. Exposure variables in this study included sociodemographic and clinical characteristics of patients, and the type of antiseptics used for wound dressing.

**Data sources/measurements:** demographic data and clinical data from the study participants were collected using a pre-tested data collection tool. Two wound swabs (before and after wound dressing) were collected from each patient, placed in Stuart´s transport media, and taken to the CUHAS Microbiology laboratory for analysis within two hours. The swabs were placed into two different sterile bottles with 2 mL of normal saline and gently shaken. Thereafter, using a standard 1μl loop, each sample was quantitatively inoculated into plain Blood agar plate and plain MacConkey agar, and incubated at 35 - 37°C for 18 - 24 hours for subsequent isolation of Gram-positive bacteria and Gram-negative bacteria, respectively. Colony-forming units (CFU) were then quantified (before and after wound dressing) under the supervision of skilled laboratory scientists and medical microbiologists [14]. To enumerate MDR bacteria, a primary culture was also done onto MacConkey agar supplemented with 2μg/ml cefotaxime and on CHROMagarMRSA® for screening of ESBL-producing Gram-negative bacteria and MRSA, respectively. Confirmation of ESBL and MRSA was done using the combined disk method and zone of inhibition of ≤ 21mm to the cefoxitin disk (30 μg), respectively [15]. Bacterial identification and MDR bacteria susceptibility testing were limited only to samples post-wound dressing.

**Bias:** potential bias was largely reduced by using quality control measures. For example, all samples were transported from the wards/clinics and processed in the laboratory within two hours, and the use of Stuart´s transport media ensured viability while limiting overgrowth of bacteria. All culture media were prepared as per the manufacturer´s instructions. Performance and sterility tests were conducted. A 0.5 McFarland standard was used to standardize bacterial inoculum density for the antimicrobial susceptibility testing for ESBL and MRSA. The standard reference strains are Methicillin-sensitive *S. aureus* (ATCC-25923), MRSA (ATCC 29213), Non-ESBL E. coli (ATCC-25922), and ESBL-*K. pneumoniae* (ATCC -700603) were used to guide all laboratory procedures [15]. To ensure that the antiseptics used were not contaminated by microorganisms, antiseptics were collected in sterile containers prior to wound dressing weekly, and cultured for bacteria and fungi. In all cases, neither bacteria nor fungi grew.

**Study size:** a minimum sample size of 180 was estimated using Kish-Leslie formula (1965) with proportions of 10.9% from Mpogoro *et al*. 2014, and 67.7% from Moremi *et al*. 2014 in the same hospital using Z of 1.96 at 95% confidence interval and a sampling error of 0.05, a sample size between 149 and 336 would suffice [8,10,16]. A total of 223 patients with septic wounds were enrolled during the study period using a convenience sampling technique. Out of these, 20 were excluded (6 were already enrolled before, 2 had their wounds already dressed prior to sample collection, and 12 did not consent to be sampled). Therefore, the final sample size was 203.

**Quantitative variables:** anatomical sites where wound swabs were taken were categorized into three (i.e. upper limbs, lower limbs, and others). The other groups were a composite sub-category to cater for multiple sites that had small frequencies. Quantitative bacterial culture was done using a calibrated 1μL loop to get bacterial colony-forming units per ml of normal saline suspension.

**Statistical methods:** all data recorded in the questionnaires were transferred to Microsoft Excel® for coding and then transferred to STATA version 13.0 software for analysis. Results were presented in percentages or proportions for categorical variables and median (interquartile range) for continuous variables. Two-sample test of proportions was used to compare the proportions of MDR (i.e. ESBL and MRSA) before and after septic wound dressing. Significant reduction of bacterial CFU load was assessed using the Two-sample Wilcoxon rank-sum (Mann-Whitney) test. All variables with a p-value of ≤0.05 on bivariate analysis were subjected to multivariate logistic regression analysis so as to assess the independent predictors for culture positivity after wound dressing using 95% CI and a cut-off value of ≤0.05.

**Ethical considerations:** ethical approval to conduct this study was sought and provided by the Joint CUHAS, BMC Research Ethics and Review Committee (CREC 1869/2021 and CREC 1873/2021). Permission to conduct this study was granted by the Director General of BMC and Heads of Surgical Departments. The importance of the study, study procedures and right to participate or withdrawal was explained to each participant in detail. Then, a written informed consent was sought from each patient above 18 years, whereas for children, parents/guardians provided permission on their behalf (however, teenagers were also requested to sign assent forms). Confidentiality and privacy were strictly ensured. Results for laboratory investigations for samples post-wound dressing were promptly communicated to the attending doctors to guide specific patients´ management

## Results

**Participants and descriptive data:** the median age (IQR) of patients involved was 29 (26 - 37) years, with the youngest and oldest participants being 1 month and 80 years, respectively. Approximately two-thirds of participants were males. Out of 203 patients, half had wounds due to road traffic accidents (RTA), and 18.2% had a previous history of antibiotic use in the past month. The most commonly used antiseptics were povidone-iodine in approximately 70.9% (Table 1).

**Table 1:** socio demographic and clinical characteristics of patients with septic wounds at BMC

Variable		Frequency (n)	Percentage (%)
**Sex**	Male	134	66.0
	Female	69	34.0
**Residency category**	Mwanza	58	28.6
	Outside Mwanza	145	71.4
**Marital status**	Married	134	66.0
	Single	69	34.0
**Education level**	None and primary	63	31.0
	Secondary	111	54.7
	College and above	29	14.3
**Occupation**	Employed	36	17.7
	Self-employed	121	59.6
	Others	46	22.7
**Referral status**	Referred	48	23.6
	Self-referral from home	155	76.4
**Cause of septic wound**	Surgical wound	44	21.7
	Traffic accident	102	50.2
	Burn injury	42	20.7
	Others*	15	7.4
**Sample site**	Upper limb	31	15.3
	Lower limb	121	59.6
	Others	51	25.1
**Antibiotic use**	Yes	37	18.2
	No	166	81.8
**Antiseptic used**	Povidone-iodine	144	70.9
	Savlon	36	17.7
	Others**	23	11.3
**Antiseptic dilution status**	Undiluted	148	72.9
	Diluted	55	27.1

*Animal bite (3), congenital (4), infection (3), cancer (1), suicide (1), perineal tear (1), cut wound (1) and unknown (1); **Normal saline mixed with other agents such as hydrogen peroxide, metronidazole or; BMC: Bugando medical centre

### Outcome data

**Figure 1:**
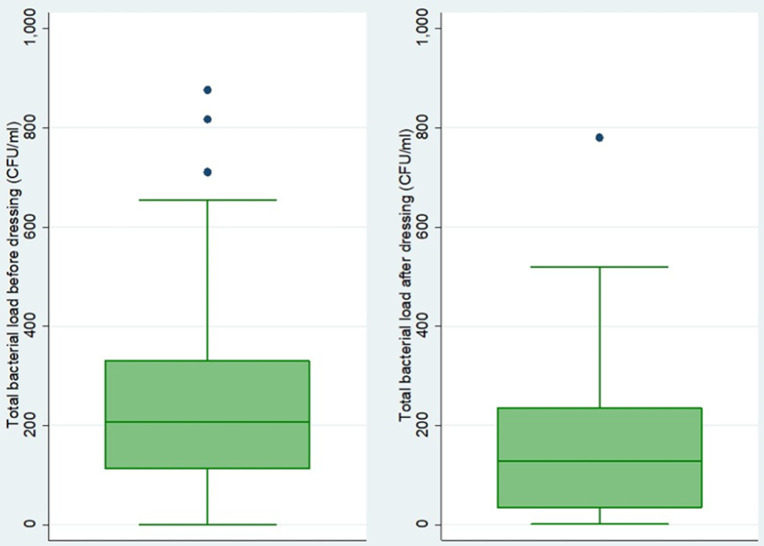
comparison of median total bacterial load before and after wound dressing

**Figure 2:**
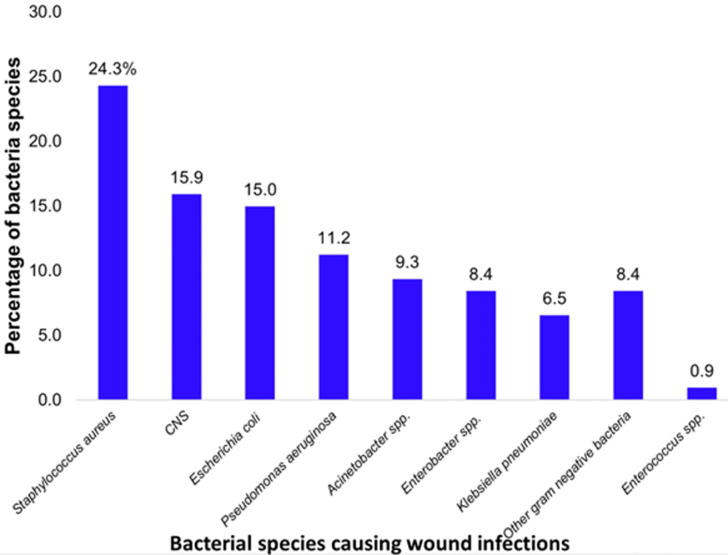
bacterial species distribution among patients with septic wounds after dressing

**The total bacterial load before and after wound dressing among patients with septic wounds:** the majority of patients were culture positive before wound dressing (71.9%, 146/203), and the culture positivity significantly decreased to 39.4% (80/203) after wound dressing (p-value < 0.001). There was a significant total median (IQR) bacterial load reduction from 205.5 (112-330) before wound dressing to 128 (34.5-235) after wound dressing (p-value < 0.001), Figure 1. The reduction was also evident for povidone-iodine [210 (112-347) to 0 (0-60) CFU/ml], Savlon [191.5 (124-295) to 96 (21-216) CFU/ml], and other antiseptic mixtures consisting of normal saline mixed with other agents such as hydrogen peroxide or metronidazole or spirit [234 (105-330) to 127 (27-205) CFU/ml]. A total of 107 bacteria were isolated from 80 positive culture swabs after wound dressing, with the most common bacterial species being *Staphylococcus aureus* (24.3%), Coagulase Negative Staphylococci (15.9%), and *Escherichia coli* (15%), Figure 2.

**Multidrug-resistant bacterial load before and after wound dressing among patients:** there was a significant decrease in the proportions of MDR bacteria after septic wound dressing among patients. Specifically, ESBL decreased from 34.0% (69/203) to 20.2% (41/203), p-value=0.0018, and MRSA decreased from 39.9% (81/203) to 11.3% (23/203), p-value<0.001. The median ESBL bacterial load reduction before and after wound dressing was 90 (40-147) CFU/ml and 40 (18-100) CFU/ml, respectively (p-value = 0.0140). On the other hand, the median (IQR) MRSA bacterial load reduction before and after wound dressing was 95 (50-167) CFU/ml and 30 (6-150) CFU/ml, respectively (p-value = 0.0169). The proportion of *Staphylococcus aureus* isolates that were confirmed to be MRSA was 88.5% (23/26), and the proportion of Gram-negative bacteria that were confirmed to produce ESBL was 68.3% (43/63).

**Factors associated with culture positivity after wound dressing:** out of 203 participants, 80 (39.4%) had aerobic culture-positive results after septic wound dressing. On bivariate analysis, the odds of culture positivity increased with education level (college and above) [OR: 95% CI = 2.83 (1.15-7.01),' p-value=0.024], and diluted antiseptics OR: 95% CI = 6.36 (3.21-12.61), p-value<0.001]. However, it decreased with povidone-iodine [OR: 95% CI = 0.08 (0.02-0.24), p-value<0.001] and antibiotic use [OR: 95% CI = 0.43 (0.19-0.97), p-value=0.042]. On multivariate logistic regression analysis, the use of povidone-iodine was independently associated with decreased odds of culture positivity after septic wound dressing [OR: 95% CI = 0.09 (0.01-0.63), p-value =0.016], Table 2.

**Table 2:** factors associated with bacterial culture positivity after wound dressing

Variable		Bacteria culture after dressing		Bivariate	Multivariate
		Negative n (%)	Positive n (%)	OR (95%CI), P-value	OR (95%CI), P-value
**Sex**	Male	84 (62.7)	50 (37.3)	1	
	Female	39 (56.5)	30 (43.5)	1.29 (0.72-2.33), 0.395	
**Residence**	Mwanza city	37(63.8)	21 (36.2)	1	
	Outside Mwanza City	86 (59.3)	59 (40.7)	1.21 (0.64-2.27), 0.555	
**Level of education**	None and primary	42 (66.7)	21 (33.3)	1	1
	Secondary	69 (62.2)	42 (37.8)	1.21 (0.64-2.33), 0.553	1.47 (0.70-3.09), 0.309
	College and above	12 (41.4)	17 (58.6)	2.83 (1.15-7.01), 0.024	1.54 (0.52-4.53), 0.431
**Referral status**	Self-referred from home	92 (59.4)	63 (40.6)	1	
	Referral	31 (64.6)	17 (35.4)	0.80 (0.41-1.57), 0.518	
**Cause of septic wound**	Surgical wound	26 (59.1)	18 (40.9)	1	
	Traffic accident	68 (66.7)	34 (33.3)	0.72 (0.35-1.50), 0.381	
	Burn injury	24 (57.1)	18 (42.9)	1.08 (0.46-2.55), 0.855	
	Others*	5 (33.3)	10 (66.7)	2.89 (0.84-9.88), 0.091	
**Sample sites**	Upper limb	17 (54.8)	14 (45.2)	1	
	Lower limb	78 (64.5)	43 (35.5)	0.67 (0.30-1.49), 0.325	
	Others	28 (54.9)	23 (45.1)	1.00 (0.41-2.45), 0.996	
**Wound duration**	1-7 days	11 (57.9)	8 (42.1)	1	
	8-30 days	92 (59.7)	62 (40.3)	0.93 (0.35-2.43), 0.877	
	>30 days	20 (66.7)	10 (33.3)	0.69 (0.21-2.25), 0.536	
**Antibiotics**	No	95 (57.2)	71 (42.8)	1	1
	Yes	28 (75.7)	9 (24.3)	0.43 (0.19-0.97), 0.042	0.65 (0.27-1.54), 0.325
**Type of antiseptics Others**	Normal saline with others	4 (17.4)	19 (82.6)	1	1
	Povidone-iodine	106 (73.6)	38 (26.4)	0.08 (0.02-0.24), <0.001	0.09 (0.01-0.63),0.016
	Savlon	13 (36.1)	23 (63.9)	0.37 (0.10-1.33), 0.129	0.33 (0.09-1.21), 0.094
**Antiseptics dilution status**	Undiluted	107 (72.3)	41 (27.7)	1	1
	Diluted	16 (29.1)	39 (70.9)	6.36 (3.21-12.61), <0.001	1.12 (0.18-6.90), 0.905

## Discussion

The majority of patients in this study were males (66%) in the middle and productive ages (i.e., second and third decades of life). The predominance of males was similar to another study at the University of Benin Teaching Hospital (58%) [17]. This can be accounted for by the fact that most of septic wounds in this study were caused by RTA, and it is well known that males are mostly involved in outdoor activities, which put them at risk of being involved in RTA, in contrast to females. However, our findings contrast with another study in Nigeria, which showed that septic wounds associated with RTA ranked fourth after surgical wounds, diabetic sores, and cancer-related wounds [5]. This shows variations in the risk exposures for wound infections across countries, which calls for country-specific responsive measures. However, our findings are reiterating the significant contribution of RTA to septic wounds, calling for focused advocacy and preventive measures involving public transport operators [18,19]. The proportion of positive aerobic cultures was high before wound dressing (71%) and was halved after dressing. *Staphylococcus aureus*, CNS, *Escherichia coli* and *Pseudomonas aeruginosa* were predominant isolates after wound dressing. The high proportion before dressing was similar to other previous studies from Benin Teaching Hospital in Nigeria (89.4%), Mulago National Hospital in Uganda (68.8%), and Muhimbili National Hospital in Tanzania (90.0%) [17,20,21]. The findings are also similar to 67.7% from patients with chronic lower limb ulcers and 72% from women with SSIs post-caesarian section at BMC conducted 7 years ago [8,10]. However, there may be slight variations in these results due to differences in patient groups, culture techniques used, and history of previous use of antibiotics. This study underscores an increase in the prevalence of MDR strains among patients with skin and soft tissue infections in this tertiary hospital, notably, ESBL-producing Gram-negative bacteria (from 13.7% in 2014 to 34.0% in the current study), and MRSA from 2.7% in 2014 to 39.9% in the current study [10]. There is also a significant increase in the proportions of ESBL among Gram-negative bacteria (from 35% in 2014 to 68.3%), and MRSA among *Staphylococcus aureus* (from 44.4% in 2014 to 88.5%) [10]. The predominance of MRSA and ESBL has also been previously reported in the same hospital among general surgical patients connoting predilection of MDR strains in the hospital settings [9]. However, the situation is different in community-associated skin and soft tissue infections in Morogoro, Tanzania, where the two MDR phenotypes are rarely found [22]. These findings highlight the fact that, although the proportions of culture positivity among patients with skin and soft tissue infections have remained relatively constant, the proportions of MDR strains in these patients are alarmingly high in the hospital settings, calling for strengthening of antimicrobial stewardship and IPC measures.

This study has shown a significant bacterial load reduction (both total bacteria, ESBL, and MRSA) after wound dressing, emphasizing the importance of this procedure in wound management prior to instituting antimicrobial therapies. However, the reduction was more with povidone-iodine than salvon and normal saline mixed with other antimicrobial agents. Povidone-iodine´s effectiveness was maintained even after controlling for potential confounders, whereby the OR and 95%CI remained protective [0.09 (0.01-0.63), p-value=0.016]. A study done in the USA showed that all antiseptics used (chlorhexidine, chlorhexidine in isopropyl alcohol, povidone-iodine, povacrylex-iodine in isopropyl alcohol, and isopropyl alcohol) were effective, with observed incidence of SSIs ranging from 4.0% to 6.0% [23]. In a systematic review involving 27 randomized controlled trials, iodine was shown to be safe and superior to silver sulfadiazine cream and non-antiseptic dressings. However, it was inferior to honey-based dressing and Rifamycin-based dressing in terms of time to complete bacterial clearance and wound healing [24]. A recent randomized controlled trial involving 54 hospitals in seven low-and-middle income countries showed similar results in preventing SSIs among patients with clean-contaminated or dirty surgical wounds when using 2% alcoholic chlorhexidine skin preparation compared with povidone-iodine, or with triclosan-coated sutures compared with non-coated sutures [13]. Therefore, these findings emphasize the usefulness of the widely available and cost-effective povidone-iodine antiseptic in wound dressing. However, the fact that diluted antiseptics had little to no effect on bacterial load reduction in bivariate analysis calls for further studies to assess staff compliance with antiseptic dilution guidelines at BMC, review of shelf-life for antiseptics, review of sources/manufacturers of antiseptics as well as evaluation of antiseptics´ active ingredients so as to institute specific remedial IPC measures.

**Limitations of the study:** firstly, this study was cross-sectional in nature conducted between May and August 2021, and therefore could not assess the trend of septic wound infections at BMC over time. Secondly, anaerobic culture was not done due to the unavailability of diagnostic infrastructures, and therefore, this may underestimate the true burden of septic wound infections. Lastly, the active ingredients in various antiseptics were not evaluated, and can be an area of focus in future studies.

## Conclusion

This study showed that approximately 70% of septic wounds were microbiologically confirmed to be infected with bacteria at BMC tertiary hospital prior to wound dressing, and this significantly decreased to 39.4% after dressing. Half these wounds were due to RTA. There was a significant reduction of the total bacteria, ESBL and MRSA load, notably by povidone-iodine. Routine monitoring of antiseptics’ effectiveness is reiterated as adjunct to antibiotics during septic wound management. Assessment of the active ingredients in the antiseptics used will be of interest in future studies. Road traffic accident victims should be prioritized in the preventive measures for MDR attributable septic wounds.

### What is known about this topic

- Septic wound dressing is a critical step in reducing bacterial load, and is a pre-requisite to facilitate bacteriostatic and/or bactericidal effects of antibiotics;
- Various types of antiseptic agents are widely used for septic wound dressing with variable effectiveness and efficiency.

### What this study adds

- Effective septic wound dressing results into a significant decrease of both pathogenic bacteria and multi-drug resistant bacteria;
- Progressive monitoring of the effects of various antiseptic agents is mandatory as part and parcel of implementing the strategic objective number three of the Tanzania National Action Plan and Global Action Plan on antimicrobial resistance.
